# Associations between prior and subsequent sickness absence before and during the COVID-19 pandemic: a Swedish prospective cohort study of 306 933 blue-collar workers in the retail and wholesale industry

**DOI:** 10.1136/bmjopen-2024-096764

**Published:** 2025-10-27

**Authors:** Lukasz Cybulski, Emma Pettersson, Kristina Alexanderson, Kristin Farrants

**Affiliations:** 1 Division of Insurance Medicine, Department of Clinical Neuroscience, Karolinska Institutet, Stockholm, Sweden

**Keywords:** COVID-19, OCCUPATIONAL & INDUSTRIAL MEDICINE, EPIDEMIOLOGY, PUBLIC HEALTH

## Abstract

**Abstract:**

**Objectives:**

The length and frequency of previous sickness absence (SA) spells have been shown to be associated with future SA. The aim was to examine if this pattern persisted during the COVID-19 pandemic among workers in retail and sales.

**Design:**

We used pseudonymised, individual-level data from three nationwide Swedish administrative registers to conduct a prospective cohort study.

**Setting:**

Sweden.

**Participants:**

All 306 933 blue-collar workers in retail and wholesale, aged 18–67 in Sweden in 2019.

**Outcomes:**

Likelihood and length of SA.

**Methods:**

We used a Negative Binomial Hurdle model to estimate incidence rate ratios (IRR) and odds ratios (ORs) to determine if SA patterns differed in 2020–2021 compared with 2018–2019. We examined how these patterns varied according to the length and frequency of SA in the preceding year. Only SA spells >14 days were included.

**Results:**

54 993 (18.5%) workers had SA during 2020–2021, an increase from 46 024 (15.6%) in 2018–2019. We observed a dose-response association between the number of prior SA days and the likelihood and length of future SA days, both before and during the pandemic. The likelihood of subsequent SA was higher in 2020–2021 compared with 2018–2019 among individuals with up to 180 prior SA days. Individuals with no prior SA had a lower average number of subsequent SA days during the pandemic (IRR (95% CI) 0.96 (0.94–0.98)) than in 2018–2019, while those with 1–30, 31–90 or 181–365 prior SA days had a higher average number of SA days during 2020–2021.

**Conclusion:**

Individuals with many SA days prior to the pandemic were at particularly high risk of lengthy SA during the pandemic years.

**STRENGTHS AND LIMITATIONS OF THIS STUDY**

- By using multiple linked population-based registers, we could delineate a fully enumerated cohort of all blue-collar workers in Sweden in the retail and wholesale sector.
- This also allowed us to obtain detailed individual-level data on sociodemographic characteristics, occupational information and labour market participation.
- Missing data was low, and there was no risk of recall bias.
- Our analyses were restricted to sickness absence spells that exceeded 14 days.

## Introduction

 Sickness absence (SA) rates increased markedly during the COVID-19 pandemic. To date, most research describing the patterns and antecedents of SA during the pandemic has focused on either the entire working population^1^,^3^ or on healthcare workers.^4^,^6^ The latter category has been of particular interest given the elevated workload and risk of exposure to COVID-19 that its workers experienced. Blue-collar workers in retail and sales share some similarities with this group. For instance, many workers in these professions were also considered indispensable during the pandemic, and those who continued to work without the possibility of doing so remotely might have experienced an increased risk of COVID-19 infection at work and when commuting to and from work. In addition, working conditions linked with higher risks of SA, including shift work,^7^ lower job control,^8 9^ higher job insecurity^10^ and exposure to specific stressors, such as repetitive movement,^11^ are also common in the retail and sales industries. For instance, prior research suggests that workers in retail and wholesale experience high rates of musculoskeletal injuries,^12^ which is one of the leading causes of SA. Moreover, shorter shift intervals in retail and sales have also been associated with higher risks for SA in this group.^13^ The effect of some of these occupational stressors might have been intensified during the pandemic and affected occupational groups unequally. For example, a Japanese analysis of 12 industries prior to and during the pandemic observed elevated levels of occupational stress among wholesale/retail and accommodation/restaurant workers.^14^ Thus, blue-collar workers in retail and wholesale may constitute an occupational category at increased risk, but little is known about SA patterns in this group before and during the pandemic.

Another factor that merits consideration is the association between prior and future SA. Studies predating the pandemic found that SA often is followed by subsequent SA^15^ and that this association appears to be influenced by the frequency and length of prior SA. More specifically, the risk for future SA appears to increase incrementally with the number of prior SA spells,^16^ and the length of prior SA seems to be associated with the length and overall likelihood of future SA.^17^ It is conceivable that this association changed during the pandemic. The aim of this study was to explore possible associations between the number of previous SA days and subsequent SA among blue-collar workers in retail and wholesale both before and during the COVID-19 pandemic and to determine if these associations changed during the pandemic.

## Methods

### Study design

A prospective population-based cohort study of blue-collar workers in Sweden was conducted, using micro data from three administrative registers. Measures of SA before (01 January 2018–31 December 2019) and during the COVID-19 pandemic (01 January 2020–31 December 2021) were compared.

### Data and study population

Individual-level pseudonymised data from three nationwide Swedish administrative registers, linked by personal identification numbers,^18^ were used to delineate the study population and construct study outcomes and covariates. Data on sociodemographic factors, income, occupation and work-related factors were obtained from Statistics Sweden’s Longitudinal Integrated Database for Health Insurance and Labor Market Studies.^19^ Information on SA and disability pension (DP) (dates and extent (part- or full-time)) was obtained from the Swedish Social Insurance Agency’s Micro Data for Analyses of Social Insurance.^20^ The Swedish Cause of Death Register^21^ was used for information on dates of death.

The study population consisted of all individuals aged 18–67 years in 2019 who were registered as living in Sweden on 31 December in both 2018 and 2019. We included all individuals who were employed by private-sector companies in the retail and wholesale industry, had occupational codes according to the Swedish Standard for Occupational Classification (SSYK) that indicated a blue-collar occupation and had income from work, parental benefits and/or SA/DP that amounted to at least 8370 SEK (ie, 75% of the necessary income level to qualify for SA benefits from the Social Insurance Agency) (n=3 08 684). Individuals were excluded from the analysis if they were not resident in Sweden when SA exposures were measured in 2017 and 2019 (n=119), or if they had full-time DP, or lacked income from work that rendered them ineligible for SA (n=1088). In addition, individuals who died in 2021 or before were excluded from the analysis (n=544).

### Public sickness absence insurance in Sweden

In Sweden, individuals with income from work, parental leave or unemployment benefits can claim SA benefits if their work capacity is reduced due to morbidity. Because individuals who are unemployed also qualify for SA benefits, there is no risk of under-recording SA if individuals become unemployed after or during a SA spell. After a first waiting day, employers pay SA benefits during the subsequent 13 days of a SA spell. Thereafter, payments are covered by the Social Insurance Agency (the Social Insurance Agency pays from day two for unemployed people). During most of the pandemic, individuals could claim reimbursement for the first SA waiting day in order to encourage people with symptoms to stay home. In this study, only information on SA spells lasting longer than 14 days was used in the computation of the outcome and exposure measures. This is because most SA spells below this duration are not recorded by the Social Insurance Agency. In the years studied, there was no maximum duration of a SA spell; it could go on for years. All residents in Sweden aged 19 to 64 years, whose work capacity is reduced permanently or long-term due to morbidity, can be granted DP from the Social Insurance Agency. SA benefits cover 80%, and DP benefits cover 64% of lost income, both up to a certain level. Both SA and DP can be granted part-time or full-time (ie, 25%, 50%, 75% or 100% of ordinary working hours). This means that people can be on part-time SA and DP at the same time, and for this reason, we calculated net days. For example, two gross days of 50% absence were combined to one net day.

### Outcomes, exposures and covariates

The outcome measure was the number of SA days in the two time periods: before the COVID-19 pandemic (01 January 2018–31 December 2019) and during the pandemic (01 January 2020–31 December 2021). The primary exposure was the number of SA days, measured the calendar year before each of these two periods, ie, in 2017 and in 2019, respectively. The exposure of SA was categorised into four groups: 0 days, 1–30, 31–90, 91–180 and 181–365. The extent of the first 14 days of a SA spell was set as the extent of day 15 of the SA spell (ie, 25%, 50%, 75% or 100%). While we could not access data on SA spells below 15 days, we nevertheless included a ‘1–30 days’ category for our SA exposure measure. This is because some SA spells started before the exposure period (eg, in 2016) and continued into the next year and then ended shortly thereafter (eg, beginning of 2017). In these cases, individuals may have had less than 14 days of SA in the exposure period (eg, 2017), but more than 15 days across both 2016 and 2017. Similarly, SA spells that began shortly before the end of an exposure period (eg, 28 December 2017) but which continued for more than 15 days into the next year will also have been in this category.

Information on the following sociodemographic variables was included as covariates in the analysis: sex; age (18–24, 25–34, 35–44, 45–54, 55–64 or 65+years); country of birth (Sweden, other Nordic country, other EU27 or rest of world and missing values (n=27, 0.0%)); educational level (elementary (0–9 years and missing values (n=2313, 0.8%)), high school (10–12 years) or university/college (>12 years)); family situation (single without children (<18 years) at home, single with children at home, married/cohabiting without children at home or married/cohabiting with children at home); place of residence (cities, towns or rural areas), according to Eurostat’s Degree of Urbanisation (DEGURBA)^22^; type of occupation based on SSYK^23^ categorised into the following 15 groups: sales assistant for daily goods, sales assistant for specialist trade, construction workers, craft workers, cashiers, warehouse and terminal staff, machine and process operators, mechanics, technicians, repair, installation etc, motor vehicle mechanics and repair personnel, transport occupations, security staff, porters, cleaners, etc, other logistics, other service staff, other sales staff or other occupations.

### Statistical analysis

We estimated the distribution of sociodemographic and occupational characteristics within the cohort. We also calculated the proportion of individuals within each SA exposure category (ie, the number of SA days in the preceding year) for both exposure periods (ie, 2017 and 2019), as well as the median and interquartile range (IQR) for the number of SA days in respective follow-up periods (ie, 2018–2019 and 2020–2021). For this last estimate, we also distinguished between those with and without SA in 2018–2019 and 2020–2021. We applied a Negative Binomial Hurdle regression to study the average number of SA days in 2018–2019 and in 2020–2021, respectively. This is a two-component model that is appropriate when analysing over-dispersed count data with an excess of zero counts^24^ (please see online supplemental material figure S1). The method divides the analysis into two separate components and allowed us to examine how prior SA was associated with the likelihood and length of future SA spells. Component (1), known as the hurdle component, makes use of a logistic regression and was applied to estimate the odds of having SA. Component (2), referred to as the count component, uses a truncated negative binomial regression and was used to estimate the average number of SA days for individuals who have had at least one SA day. Crude and adjusted (sex, age, educational level, family situation/marital status, country of birth, place of residence and occupation) odds ratios (ORs and incident rate ratios (IRRs) from the two components are presented along with 95% CIs. The exposures and covariates were modelled with a time interaction (before vs during the COVID-19 pandemic), and a robust sandwich variance/covariance matrix was used to account for repeated measures of the same individuals. To adjust for days of disability pensions during the study period, an offset^25^ was included in the model. The predicted probabilities and average number of net SA days were computed from the hurdle, count and overall model components. The overall predictions combine the predicted probabilities and average counts estimated from the two components of the model. Contrast hypothesis tests were also conducted to test for differences in the OR and IRR of SA in the 2018–2019 vs 2020–2021 period at various levels of prior SA. Analyses were performed in R V. 4.3.1 using the ‘countreg’ and ‘emmeans’ packages.

### Patient and public involvement

Results from this work will be presented directly to representatives of AFA Försäkring, the funder of this project, as well as other civil society organisations and social partners.

## Results

**Figure 1:**
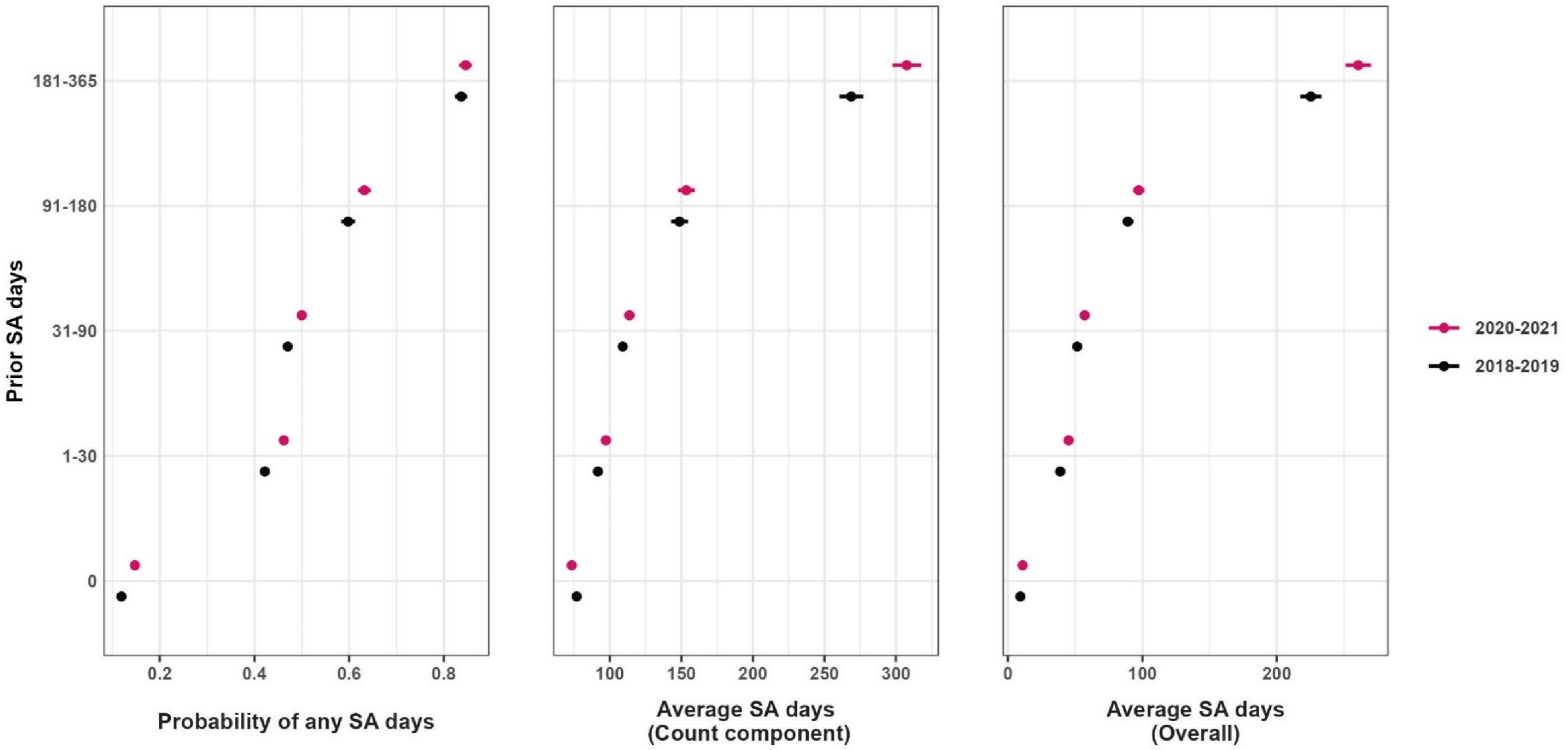
Estimated probability of sickness absence (SA) and average number of SA days (count component^a^ and overall) with 95% confidence intervals across the two study periods (2018–2019 and 2020–2021, respectively) by number of prior SA days.^a^ The count component estimates the average number of SA net days among individuals who had some SA (ie, >0 SA net days), using a truncated negative binomial model. The probability of having any SA is estimated from the hurdle component of the model, using a logistic regression model. The overall component combines estimates from the count and hurdle components, producing estimates of the average number of SA net days in the entire cohort.

Of the included 306 933 blue-collar workers in the retail and wholesale industry, 57.2% were aged 18–34 and 47.9% were women (table 1). The mean age was 35.2 (SD: 13.1). Almost a fifth (18.5%) of the cohort had at least one SA spell in the 2020–2021 period, an increase from 15.6% in 2018 (table 2). However, the number of SA days among those with SA was slightly lower during the pandemic (median (IQR: 48 (24, 107) in 2020–2021 vs 54 (28, 115) in 2018–2019) (mean number of SA days is shown in figure 1). Of those with no SA in 2019, 14.7% had at least one SA spell in the pandemic period 2020–2021; the corresponding figure for 2018–2019 was 11.9%.

**Table 1:** Cohort characteristics in 2019 among blue-collar workers in retail and wholesale

	Study cohort
Characteristic	n=3 06 933
Sex	
Female	147 127 (47.9%)
Male	159 806 (52.1%)
Age	
25–34	94 167 (30.7%)
18–24	81 320 (26.5%)
35–44	50 602 (16.5%)
45–54	45 205 (14.7%)
55–64	31 823 (10.4%)
65+	3 816 (1.2%)
Educational level	
College/university	56 794 (18.5%)
High school	206 934 (67.4%)
Elementary	40 892 (13.3%)
Missing	2 313 (0.8%)
Family situation	
Single without children (<18 years) at home	185 315 (60.4%)
Single with children at home	10 622 (3.5%)
Married/cohabiting without children at home	36 777 (12.0%)
Married/cohabiting with children at home	74 219 (24.2%)
Region of birth	
Sweden	259 521 (84.6%)
Nordic countries (except Sweden)	3 273 (1.1%)
EU27 (except Denmark, Finland or Sweden)	7 651 (2.5%)
Rest of the world	36 461 (11.9%)
Missing	27 (0.0%)
Type of place of residence^*^	
Large city	120 211 (39.2%)
Medium-size town	130 722 (42.6%)
Small town or rural area	56 000 (18.2%)
Occupation	
Sales assistant for specialist trade	96 607 (31.5%)
Sales assistant for daily goods	80 113 (26.1%)
Warehouse and terminal staff	35 660 (11.6%)
Motor vehicle mechanics and repair personnel	22 638 (7.4%)
Other service staff	13 613 (4.4%)
Mechanics, technicians, repair, installation, etc	11 772 (3.8%)
Security staff, porters, cleaners, etc	7 056 (2.3%)
Construction workers	6 370 (2.1%)
Other sales staff	6 306 (2.1%)
Other logistics	6 246 (2.0%)
Cashiers	6 215 (2.0%)
Transport occupations	4 712 (1.5%)
Machine and process operators	3 654 (1.2%)
Craft workers	3 262 (1.1%)
Other occupations	2 709 (0.9%)

* Defined according to Eurostat’s Degree of Urbanisation (DEGURBA) (22).

**Table 2:** Sickness absence among blue-collar workers in the periods 2020–2021 and 2018–2019, respectively, and by prior sickness absence (SA) days among 306 933 blue-collar workers in retail and wholesale

2020–2021			
	Proportion with any SA net days	SA net days (among individuals with any SA)	SA net days (among all in the cohort)
	N (%)	Median (IQR)	Median (IQR)
Overall	54 993 (18.5%)	48 (24, 107)	0 (0, 0)
Prior SA days 2019			
0	39 550 (14.7%)	42 (22, 87)	0 (0, 0)
1–30	4 442 (46.2%)	53 (26, 120)	0 (0, 47)
31–90	5 605 (50.0%)	65 (29, 144)	1 (0, 65)
91–180	3 038 (63.3%)	91 (40, 199)	33 (0, 124)
181–365	2 358 (84.7%)	229 (78, 485)	161 (32, 413)

2018–2019			
	Proportion with any SA net days	SA net days (among individuals with any SA)	SA net days (among all)
	N (%)	Median (IQR)	Median (IQR)
Overall	46 024 (15.6%)	54 (28, 115)	0 (0, 0)
Prior SA days 2017			
0	31 980 (11.9%)	47 (27, 93)	0 (0, 0)
1–30	3 942 (42.2%)	52 (25, 116)	0 (0, 40)
31–90	4 957 (47.1%)	66 (31, 143)	0 (0, 61)
91–180	2 589 (60.0%)	95 (37, 198)	26 (0, 120)
181–365	2 556 (83.7%)	207 (84, 396)	154 (32, 365)

The hurdle component of the negative binomial regression model showed that individuals with a higher number of prior SA days were more likely to have subsequent SA compared with those with no prior SA (table 3). These associations were observed both in the 2018–2019 and the 2020–2021 periods, although the associations were attenuated somewhat during the pandemic. Among those with no prior SA (the reference group), there was an increase in the likelihood of having SA in 2020–2021 compared with in 2018–2019 (as indicated in table 4, OR (95% CI) 1.29 (1.27–1.31)). mong individuals with 0, 1–30, 31–90 or 91–180 prior SA days, the likelihood of having subsequent SA was higher in 2020–2021, compared with 2018–2019. However, the likelihood of subsequent SA among individuals with 181–365 prior SA days was not significantly different in the two periods (OR (95% CI) 1.07 (0.93–1.23)).

**Table 3:** Incident rate ratios (IRRs) and ORs showing the crude and adjusted associations between prior sickness absence days, sociodemographic factors and occupation with the likelihood and length of sickness absences in days in the two periods 2018–2019 and 2020–2021, respectively, among 306 933 blue-collar workers in retail and sales

	Count component^*^		Hurdle component^†^	
	2018–2019	2020–2021	2018–2019	2020–2021
Characteristic	IRR (95% CI)	IRR (95% CI)	OR (95% CI)	OR (95% CI)
Prior number of sickness absence days^‡^				
		Crude models		
0	—	—	—	—
1–30	1.19 (1.15–1.24)	1.32 (1.19–1.47)	5.39 (5.17–5.63)	4.96 (4.41–5.58)
31–90	1.42 (1.37–1.47)	1.55 (1.41–1.70)	6.57 (6.31–6.83)	5.78 (5.18–6.44)
91–180	1.93 (1.86–2.02)	2.09 (1.87–2.34)	10.99 (10.33–11.69)	9.94 (8.38–11.78)
181–365	3.50 (3.38–3.62)	4.19 (3.83–4.59)	37.92 (34.42–41.77)	31.82 (24.05–42.09)
Prior number of sickness absence days^‡^		Adjusted models^§^		
0	—	—	—	—
1–30	1.15 (1.10–1.19)	1.28 (1.15–1.42)	4.31 (4.12–4.51)	4.21 (3.73–4.76)
31–90	1.36 (1.32–1.41)	1.50 (1.37–1.65)	5.27 (5.05–5.49)	4.97 (4.43–5.56)
91–180	1.83 (1.76–1.91)	2.01 (1.79–2.26)	8.57 (8.03–9.14)	8.26 (6.93–9.85)
181–365	3.26 (3.15–3.37)	3.97 (3.62–4.35)	30.06 (27.23–33.19)	25.28 (19.01–33.62)

* The count component estimates the average number of SA net days, among individuals who had some SA (ie, >0 SA net days), using a truncated negative binomial model.

† The hurdle component estimates the odds of having any SA, using a logistic regression model.

‡ Prior SA net days were measured in 2019 for the 2020–2021 period and in 2017 for the 2018–2019 period.

§ Models adjusted for sex, age, educational level, family situation/marital status, country of birth, place of residence and occupation.

**Table 4:** Contrasts comparing estimated odds of sickness absence (SA) and average number of SA days in the periods 2020–2021 and 2018–2019, across number of prior SA days among 306 933 blue-collar workers in retail and sales

	2020–2021 versus 2018–2019	
	**Count component** ^*^	**Hurdle component** ^**†**^
	IRR (95% CI)	OR (95% CI)
Prior SA net days^‡^		
0	0.96 (0.94–0.98)	1.29 (1.27–1.31)
1–30	1.07 (1.02–1.12)	1.23 (1.16–1.30)
31–90	1.06 (1.01–1.10)	1.18 (1.11–1.24)
91–180	1.05 (0.99–1.10)	1.21 (1.11–1.32)
181–365	1.15 (1.11–1.20)	1.07 (0.93–1.23)

* The count component estimates the average number of SA net days, amongst individuals who had some SA (ie, >0 SA net days), using a truncated negative binomial model.

† The hurdle component estimates the odds of having any SA, using a logistic regression model.

‡ Prior SA net days were measured in 2019 for the 2020–2021 period and in 2017 for the 2018–2019 period.

The count component of the analysis indicated that having a higher number of prior SA days was associated with having more SA days in subsequent periods, in comparison with individuals with no prior SA. This association was observed both before and during the pandemic; however, in contrast to the results from the hurdle component, these associations were somewhat stronger during the pandemic and especially for those with a higher number of prior SA days (IRR (95% CI) 181–365 days: 3.26 (3.15–3.37) before versus 3.97 (3.62–4.36) during). These results are illustrated in figure 1, which shows that the estimated average numbers of SA days from both the count component and in the overall measure were higher during the pandemic than before, among individuals who had prior SA.

As can be seen in table 4, individuals with no prior SA had a somewhat lower average number of subsequent SA days during 2020–2021 compared with during 2018–2019 (IRR (95% CI) 0.96 (0.94–0.98)), while those with 1–30, 31–90 and 181–365 prior SA days had a higher average number of subsequent SA days during the pandemic, though no significant differences were found among those with 91–180 prior SA days. Online supplemental table S1 shows the distribution of cohort characteristics according to prior SA. Occupation and sociodemographic factors were generally less strongly associated with the duration and likelihood of SA (online supplemental table S2 and S3).

## Discussion

In this population-based prospective cohort study, we used linked micro data to examine associations between prior SA length and future SA among blue-collar workers in retail and wholesale in the years before and during the COVID-19 pandemic. To our knowledge, this has not been studied before. We observed an overall increase in the number of SA days and in the number of people with at least one SA spell during the pandemic compared with previous years. Our findings also indicate a dose-response association between prior SA length and the likelihood and length of future SA that appeared to change during the first 2 years of the pandemic.

 We found that individuals who had experienced longer SA from work were more likely to have future SA, which is consistent with previous studies.^17 26^ Moreover, these associations appeared to change somewhat during the pandemic. For instance, having any prior SA was associated with a greater likelihood of future SA during 2020–2021 than in 2018–2019, except in the group that had SA spanning 181–365 days, in whom no change was observed between these two periods. This latter group had, on average, 300 or more SA days across 2020–2021, which may have reduced their exposure to some of the pandemic-specific factors that potentially underpinned the rise in SA among individuals who had shorter SA in the preceding year and thus spent more time at work during the pandemic. For instance, Sweden did not implement a lockdown and most workers in trade and transport occupations were not furloughed and did not work remotely. At the same time, Sweden experienced relatively high rates of infection in the earlier stages of the pandemic when compared with neighbouring countries^27^ and vaccination was not widely available for the majority of workers included in our study until the second quarter of 2021.^28^ Indeed, statistics from the Swedish Social Insurance Agency show that the rise of SA spells during 2020–2021 primarily was accounted for by short SA spells due to COVID-19, with SA due to other causes being relatively stable.^29^ Taken together, this suggests that the risks for COVID-19 infection and consequent SA may have been higher among workers who spent more time at work during the pandemic, and this may explain why the association between prior SA length and likelihood of future SA was weaker among those with the longest prior SA.

In addition to COVID-19, there are also other factors that could have influenced the likelihood of SA during the pandemic. For example, the absence of a strict lockdown, which in some countries was associated with a decrease in mental well-being^30^ and which itself is a risk factor for SA, may have contributed to relatively stable rates of SA due to mental health-related diagnoses during the pandemic. Whether this influenced the likelihood of SA among workers with varying lengths of prior SA is nevertheless unclear. The pandemic also coincided with sweeping changes within workplaces that also may have influenced SA risks during this period. For instance, a recent survey showed that most respondents from the trade and retail industry experienced increased stress and workload,^31^ which outside of the pandemic has been linked to increased SA risks. It is conceivable that the influence of some of these factors was less pronounced among workers with longer prior SA who stayed at home for longer periods during the pandemic, but there is little concrete support for this. Factors other than COVID-19 have generally not been studied in relation to SA, and whether changes to working conditions or other measures implemented during the pandemic (eg, closing of schools) increased the risk for SA remains to be determined. Nevertheless, it is worth noting that the majority (81.5%) of blue-collar workers in our study had no SA spells longer than 14 days during the pandemic.

The results followed a slightly different pattern when we examined the association between the number of prior SA days and the number of SA days in the following year. More specifically, workers with longer prior SA tended to experience longer future SA, and vice versa, a finding consistent with prior research,^26^ although there were some exceptions to this in our study. As with the association between prior SA length and the overall likelihood of future SA, the strength of this association appeared to change during the pandemic, but the direction of the change varied with the length of prior SA. For example, individuals with no prior SA generally had fewer SA days during the pandemic compared with previous years. This could be because this category was qualitatively different during the pandemic relative to previous years. For example, it is possible that this group also consisted of individuals who generally are not sickness absent but who experienced incident SA spells due to COVID-19.^1^ If so, then one might expect a larger proportion of SA spells to end more quickly in this group, given that SA due to COVID-19 for many workers tended to be of shorter duration than SA due to many other diagnoses,^1^ such as musculoskeletal or mental diagnoses, CVD or cancer.^32^ There is some support for this interpretation since the rise in the proportion of SA spells during 2020–2021 was primarily driven by an increase of short SA spells due to COVID-19 among all insured.^29^ By contrast, SA spells tended to be longer during the pandemic among those who had some prior SA, except among individuals with 91–180 prior SA days, in whom there was no change between the two studied periods. However, these differences were generally small. For instance, the reduction in SA days among those with no prior SA amounted to 4% from 2018 to 2019 to 2020–2021. The exception to this pattern was the group with the highest number of prior SA days (ie, 181–365 days), among whom we observed a 15% increase in SA days. This could partly reflect legislative changes that occurred during this period. Typically, individuals are evaluated throughout their SA spell to determine if they continue to meet the criteria for sickness absence. Between December 2020 and March 2021, the Swedish government temporarily suspended assessments of on-going SA spells that had lasted between 181–365 days, which meant that a smaller proportion of SA spells ended on day 180.^29^ Longer waiting times for investigations and treatments in healthcare might also have led to longer SA spells.^29^

## Strength and limitations

A strength of this study is that we delineated a fully enumerated cohort of all blue-collar workers in Sweden employed by companies in the retail and wholesale industries. Thus, our results are not an approximation as our data refer to all workers that were employed in Sweden during all of 2019. Moreover, the data linkage used in this study allowed us to incorporate a rich array of sociodemographic and occupational information at the individual level into our analyses. Because these data are derived from routinely collected administrative data, there is also no attrition or risk for recall bias. Our study also had some limitations. For instance, we could only study SA spells that exceed 14 days, as spells below this duration are outside the purview of the Social Insurance Agency. While these longer spells account for the majority of the total number of SA days in the population, many SA spells are shorter.^33^ Thus, SA spells that lasted for less than 15 days will not have been included in this study. While our study demonstrated only a slight increase in SA during the pandemic, it is conceivable that the pattern might have been different for shorter spells. Finally, due to the observational nature of this study, the results cannot be used to infer causality.

## Conclusion

Despite the increase in morbidity and consequent disruption of the labour market during the COVID-19 pandemic, most blue-collar workers in the retail and wholesale industries had no SA during 2020–2021. While this is an encouraging finding, there was nevertheless a rise in SA during this period. Most investigations on SA during the pandemic have to date focused on examining COVID-19 as the main cause for these increases.^1^,^334 35^ Although the virus undoubtedly played a significant role, the pandemic coincided with numerous other changes which currently are poorly understood within the context of SA. For instance, workplace adaptations, organisational restructuring, anxieties around job security, or shifting priorities within healthcare services may all have influenced the likelihood or duration of SA spells during the pandemic and affected individuals with a varying degree of prior SA differently, but currently these phenomena have not been studied in relation to SA. Research is also needed to understand the long-term consequences of the pandemic. For example, research on white-collar workers in Sweden suggests that SA levels have returned to pre-pandemic levels following a rise during 2020–2021.^36^ Whether the same is true for blue-collar workers is unclear.

## Supplementary material

10.1136/bmjopen-2024-096764 online supplemental file 1

## Data Availability

Data may be obtained from a third party and are not publicly available.
