# Supplementary material for: Associations between prior and subsequent sickness absence before and during the COVID-19 pandemic: a Swedish prospective cohort study of 306 933 blue-collar workers in the retail and wholesale industry
Source: BMJ Open. 2025 Oct 27;15(10):e096764. doi: 10.1136/bmjopen-2024-096764 (PMC12581036; doi:10.1136/bmjopen-2024-096764)
Supplement: online supplemental file 1 [file bmjopen-15-10-s001.docx]

**Figure S1.** Distribution of observations according to the number of net SA days by observation period.


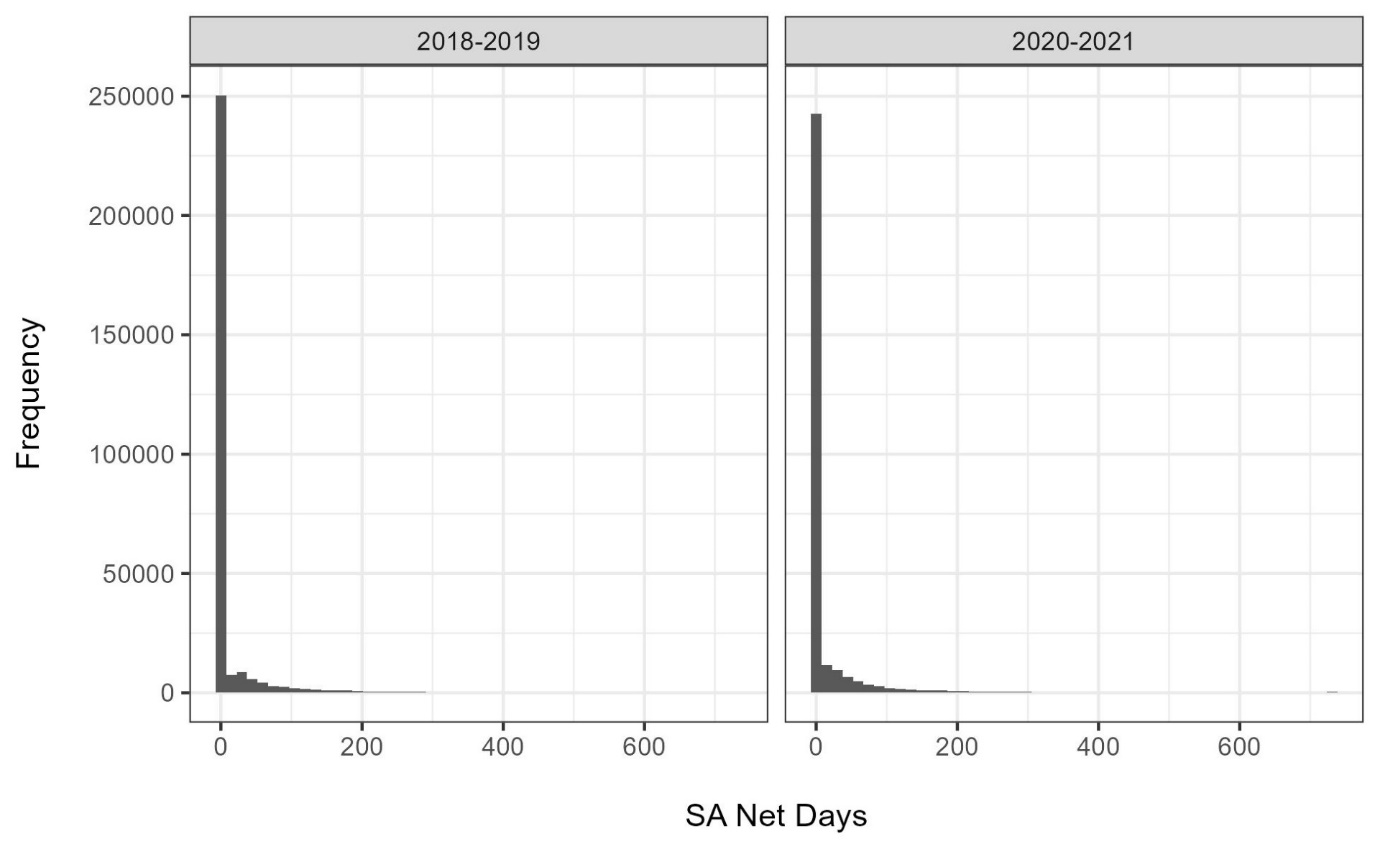


| **Table S1**. Distribution of cohort characteristics of 306,933 blue collar workers in retail and wholesale in the periods 2020-2021 and 2018-2019, overall and by prior sickness absence (SA) days | | | | | | |
| --- | --- | --- | --- | --- | --- | --- |
|  |  | **2020-2021** |  |  | **2018-2019** |  |
|  | **Proportion with any SA net days** | **SA net days  (Amongst individuals with any SA)** | **SA net days  (Amongst all in cohort)** | **Proportion with any SA net days** | **SA net days  (Amongst individuals with any SA)** | **SA net days  (Amongst all in cohort)** |
|  | **N (%)** | **Mean (SD)** | **Mean (SD)** | **N (%)** | **Mean (SD)** | **Mean (SD)** |
| **Overall** | 54 993 (18.5%) | 93 (119) | 17 (63) | 46 024 (15.6%) | 96 (113) | 15 (57) |
| **Prior SA days** |  |  |  |  |  |  |
| 0 | 39 550 (14.7%) | 74 (86) | 11 (42) | 31 980 (11.9%) | 77 (83) | 9 (38) |
| 1-30 | 4 442 (46.2%) | 95 (113) | 44 (90) | 3 942 (42.2%) | 90 (102) | 38 (80) |
| 30-90 | 5 605 (50.0%) | 112 (128) | 56 (107) | 4 957 (47.1%) | 108 (116) | 51 (96) |
| 90-180 | 3 038 (63.3%) | 149 (157) | 94 (144) | 2 589 (60.0%) | 144 (146) | 87 (133) |
| 180-365 | 2 358 (84.7%) | 295 (243) | 250 (248) | 2 556 (83.7%) | 264 (211) | 221 (217) |
| **Occupation** |  |  |  |  |  |  |
| Sales assistant for specialist trade | 16 193 (17.4%) | 93 (119) | 16 (61) | 13 792 (14.8%) | 96 (114) | 14 (56) |
| Sales assistant for daily goods | 14 363 (18.5%) | 96 (123) | 18 (65) | 12 035 (15.7%) | 98 (119) | 15 (59) |
| Warehouse and terminal staff | 8 085 (23.4%) | 93 (121) | 22 (71) | 6 445 (18.7%) | 94 (113) | 18 (61) |
| Motor vehicle mechanics and repair personnel | 4 136 (18.7%) | 86 (108) | 16 (57) | 3 329 (15.1%) | 85 (98) | 13 (49) |
| Other service staff | 2 005 (15.8%) | 85 (110) | 13 (54) | 1 697 (13.3%) | 92 (102) | 12 (48) |
| Mechanics, technicians, repair, installation, etc | 1 998 (17.5%) | 89 (111) | 16 (58) | 1 769 (15.3%) | 92 (109) | 14 (54) |
| Security staff, porters, cleaners, etc | 1 233 (18.5%) | 94 (121) | 17 (63) | 1 044 (15.8%) | 95 (107) | 15 (55) |
| Construction workers | 1 184 (19.2%) | 92 (112) | 18 (61) | 985 (16.0%) | 92 (107) | 15 (55) |
| Other sales staff | 1 045 (17.2%) | 104 (136) | 18 (69) | 897 (14.9%) | 105 (126) | 16 (62) |
| Other logistics | 1 024 (17.0%) | 87 (116) | 15 (58) | 862 (14.3%) | 93 (111) | 13 (53) |
| Cashiers | 1 072 (18.0%) | 97 (125) | 17 (65) | 904 (15.4%) | 102 (118) | 16 (59) |
| Transport occupations | 934 (20.5%) | 89 (106) | 18 (60) | 785 (17.3%) | 95 (106) | 16 (57) |
| Machine and process operators | 617 (17.6%) | 88 (109) | 16 (57) | 530 (15.0%) | 93 (111) | 14 (54) |
| Craft workers | 606 (19.2%) | 100 (118) | 19 (65) | 527 (16.8%) | 101 (113) | 17 (60) |
| Other occupations | 498 (19.2%) | 98 (131) | 19 (69) | 423 (16.4%) | 98 (115) | 16 (59) |
| **Sex** |  |  |  |  |  |  |
| Female | 29 742 (21.0%) | 97 (123) | 20 (69) | 26 201 (18.5%) | 99 (117) | 18 (63) |
| Male | 25 251 (16.3%) | 88 (114) | 14 (56) | 19 823 (12.9%) | 91 (108) | 12 (49) |
| **Age** |  |  |  |  |  |  |
| 25-34 | 16 451 (18.0%) | 88 (111) | 16 (58) | 14 266 (15.7%) | 86 (98) | 13 (50) |
| 18-24 | 7 759 (9.9%) | 72 (89) | 7 (35) | 5 217 (6.9%) | 67 (74) | 5 (26) |
| 35-44 | 10 762 (21.7%) | 96 (126) | 21 (71) | 9 073 (18.4%) | 105 (125) | 19 (67) |
| 45-54 | 11 098 (24.9%) | 100 (127) | 25 (77) | 9 125 (20.5%) | 107 (126) | 22 (72) |
| 55-64 | 8 639 (28.4%) | 109 (135) | 31 (87) | 7 705 (24.4%) | 108 (125) | 26 (77) |
| 65+ | 284 (10.0%) | 74 (79) | 7 (33) | 638 (16.9%) | 100 (113) | 17 (59) |
| **Educational level** |  |  |  |  |  |  |
| College/university | 7 847 (14.5%) | 85 (113) | 12 (52) | 6 067 (11.1%) | 90 (107) | 10 (46) |
| High school | 37 892 (18.8%) | 92 (117) | 17 (62) | 32 165 (16.0%) | 94 (112) | 15 (57) |
| Elementary | 9 254 (22.5%) | 104 (131) | 23 (76) | 7 792 (19.6%) | 105 (121) | 21 (68) |
| **Family situation** |  |  |  |  |  |  |
| Single without children (<18 years) at home | 27 790 (15.6%) | 92 (119) | 14 (58) | 21 583 (12.2%) | 95 (114) | 12 (51) |
| Single with children at home | 3 020 (29.3%) | 110 (135) | 32 (89) | 2 741 (26.5%) | 114 (130) | 30 (84) |
| Married/cohabiting without children at home | 8 987 (25.6%) | 98 (123) | 25 (76) | 7 728 (21.4%) | 102 (119) | 22 (69) |
| Married/cohabiting with children at home | 15 196 (20.9%) | 88 (113) | 18 (63) | 13 972 (19.3%) | 89 (104) | 17 (58) |
| **Region of birth** |  |  |  |  |  |  |
| Sweden | 44 796 (17.8%) | 94 (120) | 17 (62) | 38 692 (15.3%) | 96 (114) | 15 (57) |
| Norden (except Sweden) | 737 (23.8%) | 109 (132) | 26 (79) | 633 (20.4%) | 98 (116) | 20 (66) |
| EU27 (except Denmark, Finland, & Sweden) | 1 480 (20.3%) | 87 (117) | 18 (63) | 1 181 (17.0%) | 89 (106) | 15 (55) |
| Rest of the world | 7 980 (22.8%) | 85 (113) | 19 (65) | 5 518 (16.6%) | 91 (108) | 15 (56) |
| **Type of place of residence**^a^ |  |  |  |  |  |  |
| Large city | 20 889 (18.1%) | 91 (117) | 16 (61) | 16 622 (14.4%) | 95 (113) | 14 (54) |
| Medium-size town | 23 699 (18.7%) | 93 (120) | 17 (63) | 19 999 (15.9%) | 95 (113) | 15 (57) |
| Small town or rural area | 10 405 (19.1%) | 96 (120) | 18 (65) | 9 403 (17.3%) | 97 (114) | 17 (60) |
| ^a^ Defined according to Eurostat’s Degree of Urbanisation (DEGURBA) (22) | | | | | | |

| **Table S2** Crude incident rate ratios (IRRs) and odds ratios (OR) showing the associations between sociodemographic factors and occupation with the likelihood and length of sickness absences in days in the two periods 2018-2019 and 2020-2021, respectively | | | | |
| --- | --- | --- | --- | --- |
|  | **Count component^a^** | | **Hurdle component^b^** | |
|  | **2018-2019** | **2020-2021** | **2018-2019** | **2020-2021** |
| **Characteristic** | **IRR (95% CI)** | **IRR (95% CI)** | **OR (95% CI)** | **OR (95% CI)** |
| **Occupation** |  |  |  |  |
| Sales assistant for specialist trade | — | — | — | — |
| Sales assistant for daily goods | 1.01 (0.98-1.04) | 1.03 (0.96-1.11) | **1.07 (1.04-1.10)** | **1.08 (1.01-1.15)** |
| Warehouse and terminal staff | 0.97 (0.93-1.00) | 0.99 (0.92-1.08) | **1.33 (1.28-1.37)** | **1.45 (1.35-1.56)** |
| Motor vehicle mechanics and repair personnel | **0.87 (0.83-0.91)** | 0.91 (0.82-1.02) | 1.02 (0.98-1.07) | 1.09 (0.99-1.20) |
| Other service staff | **0.94 (0.88-0.99)** | 0.90 (0.78-1.05) | **0.88 (0.84-0.93)** | 0.89 (0.79-1.01) |
| Mechanics, technicians, repair, installation, etc | **0.94 (0.88-1.00)** | 0.95 (0.81-1.10) | 1.04 (0.99-1.10) | 1.01 (0.89-1.15) |
| Security staff, porters, cleaners, etc | 0.98 (0.91-1.06) | 1.01 (0.84-1.22) | **1.08 (1.01-1.16)** | 1.08 (0.92-1.27) |
| Construction workers | 0.94 (0.87-1.02) | 0.97 (0.80-1.18) | **1.10 (1.03-1.18)** | 1.13 (0.96-1.34) |
| Other sales staff | **1.10 (1.01-1.19)** | 1.13 (0.93-1.37) | 1.01 (0.94-1.09) | 0.99 (0.84-1.16) |
| Other logistics | 0.96 (0.88-1.05) | 0.94 (0.77-1.16) | 0.96 (0.89-1.03) | 0.98 (0.82-1.16) |
| Cashiers | 1.07 (0.99-1.16) | 1.06 (0.88-1.28) | 1.05 (0.97-1.13) | 1.04 (0.89-1.23) |
| Transport occupations | 0.98 (0.90-1.06) | 0.95 (0.78-1.15) | **1.21 (1.11-1.30)** | **1.23 (1.02-1.48)** |
| Machine and process operators | 0.95 (0.86-1.06) | 0.95 (0.74-1.21) | 1.02 (0.93-1.12) | 1.02 (0.81-1.27) |
| Craft workers | 1.04 (0.94-1.15) | 1.06 (0.82-1.38) | **1.16 (1.06-1.28)** | 1.13 (0.91-1.42) |
| Other occupations | 1.02 (0.91-1.14) | 1.06 (0.80-1.40) | **1.13 (1.02-1.26)** | 1.13 (0.88-1.46) |
| **Sex** |  |  |  |  |
| Female | — | — | — | — |
| Male | **0.91 (0.89-0.94)** | **0.89 (0.85-0.94)** | **0.65 (0.64-0.67)** | **0.73 (0.70-0.77)** |
| **Age** |  |  |  |  |
| 25-34 | — | — | — | — |
| 18-24 | **0.78 (0.76-0.81)** | **0.81 (0.75-0.89)** | **0.40 (0.38-0.41)** | **0.50 (0.46-0.54)** |
| 35-44 | **1.24 (1.20-1.27)** | **1.11 (1.03-1.20)** | **1.21 (1.18-1.25)** | **1.26 (1.18-1.35)** |
| 45-54 | **1.28 (1.24-1.32)** | **1.19 (1.10-1.28)** | **1.38 (1.34-1.42)** | **1.51 (1.41-1.62)** |
| 55-64 | **1.32 (1.28-1.37)** | **1.31 (1.21-1.42)** | **1.74 (1.68-1.79)** | **1.80 (1.68-1.94)** |
| 65-67 | **1.19 (1.09-1.31)** | 0.84 (0.62-1.13) | 1.09 (1.00-1.19) | **0.51 (0.39-0.66)** |
| **Educational level** |  |  |  |  |
| College/university | — | — | — | — |
| High school | **1.06 (1.03-1.10)** | **1.08 (1.00-1.17)** | **1.52 (1.47-1.56)** | **1.37 (1.28-1.47)** |
| Elementary | **1.19 (1.15-1.24)** | **1.24 (1.12-1.36)** | **1.94 (1.87-2.02)** | **1.71 (1.58-1.86)** |
| **Family situation** |  |  |  |  |
| Single without children (<18 years) at home | — | — | — | — |
| Single with children at home | **1.19 (1.14-1.25)** | **1.19 (1.07-1.33)** | **2.59 (2.47-2.71)** | **2.24 (2.02-2.50)** |
| Married/cohabiting without children at home | **1.10 (1.07-1.14)** | **1.09 (1.01-1.17)** | **1.95 (1.90-2.01)** | **1.87 (1.74-2.00)** |
| Married/cohabiting with children at home | **0.93 (0.90-0.95)** | **0.93 (0.88-0.99)** | **1.72 (1.68-1.76)** | **1.44 (1.36-1.52)** |
| **Region of birth** |  |  |  |  |
| Sweden | — | — | — | — |
| Norden (except Sweden) | 1.03 (0.94-1.14) | 1.18 (0.94-1.49) | **1.41 (1.29-1.54)** | **1.44 (1.18-1.76)** |
| EU27 (except Denmark, Finland, & Sweden) | **0.91 (0.85-0.97)** | 0.92 (0.79-1.08) | **1.13 (1.06-1.20)** | **1.18 (1.02-1.36)** |
| Rest of the world | **0.93 (0.90-0.96)** | **0.89 (0.82-0.96)** | **1.10 (1.07-1.13)** | **1.36 (1.27-1.46)** |
| **Type of place of residence^c^** |  |  |  |  |
| Large city | — | — | — | — |
| Medium-size town | 1.00 (0.98-1.03) | 1.04 (0.98-1.10) | **1.12 (1.09-1.14)** | 1.04 (0.99-1.10) |
| Small town or rural area | **1.03 (1.00-1.06)** | 1.07 (0.99-1.15) | **1.24 (1.21-1.28)** | **1.07 (1.00-1.14)** |
| ^a^ The Count component estimates the average number of SA net days, amongst individuals who had some SA (i.e., >0 SA net days), using a truncated negative binomial model. ^b^ The Hurdle component estimates the odds of having any SA, using a logistic regression model. ^c^ Defined according to Eurostat’s Degree of Urbanisation (DEGURBA) (22) | | | | |

| **Table S3**. Adjusted incident rate ratios (IRRs) and odds ratios (OR) showing the associations between sociodemographic factors and occupation with the likelihood and length of sickness absences in days in the two periods 2018-2019 and 2020-2021, respectively | | | | | |
| --- | --- | --- | --- | --- | --- |
|  | **Count component^a^** | | **Hurdle component^b^** | |  |
|  | **2018-2019** | **2020-2021** | **2018-2019** | **2020-2021** |  |
| **Characteristic** | **IRR (95% CI)** | **IRR (95% CI)** | **OR (95% CI)** | **OR (95% CI)** |  |
| **Occupation** |  |  |  |  |  |
| Sales assistant for specialist trade | — | — | — | — |  |
| Sales assistant for daily goods | 1.01 (0.99-1.04) | 1.00 (0.93-1.08) | **1.05 (1.02-1.08)** | 1.05 (0.97-1.14) |  |
| Warehouse and terminal staff | 0.99 (0.96-1.02) | 1.00 (0.91-1.10) | **1.47 (1.42-1.52)** | **1.46 (1.33-1.62)** |  |
| Motor vehicle mechanics and repair personnel | **0.94 (0.90-0.98)** | 0.99 (0.88-1.11) | **1.29 (1.23-1.35)** | **1.26 (1.11-1.43)** |  |
| Other service staff | 1.00 (0.95-1.06) | 0.94 (0.81-1.09) | 1.02 (0.96-1.08) | 0.99 (0.84-1.15) |  |
| Mechanics, technicians, repair, installation, etc | 0.98 (0.93-1.03) | 1.01 (0.86-1.18) | **1.28 (1.20-1.36)** | 1.14 (0.97-1.34) |  |
| Security staff, porters, cleaners, etc | 1.00 (0.94-1.07) | 1.06 (0.87-1.29) | **1.10 (1.02-1.19)** | 1.06 (0.87-1.30) |  |
| Construction workers | 1.01 (0.94-1.08) | 1.04 (0.85-1.26) | **1.31 (1.21-1.42)** | 1.22 (0.98-1.50) |  |
| Other sales staff | **1.11 (1.03-1.19)** | 1.09 (0.89-1.34) | **1.10 (1.02-1.19)** | 1.04 (0.85-1.28) |  |
| Other logistics | 1.04 (0.96-1.13) | 0.94 (0.76-1.16) | 1.07 (0.99-1.16) | 1.01 (0.82-1.25) |  |
| Cashiers | 1.06 (0.99-1.14) | 1.02 (0.83-1.25) | 1.00 (0.93-1.09) | 1.04 (0.84-1.28) |  |
| Transport occupations | 1.03 (0.96-1.11) | 0.99 (0.81-1.22) | **1.39 (1.28-1.52)** | **1.30 (1.02-1.64)** |  |
| Machine and process operators | 0.96 (0.87-1.05) | 0.97 (0.75-1.26) | 1.11 (1.00-1.23) | 1.03 (0.78-1.35) |  |
| Craft workers | 1.07 (0.97-1.17) | 1.11 (0.85-1.45) | **1.20 (1.08-1.33)** | 1.11 (0.84-1.47) |  |
| Other occupations | 1.03 (0.93-1.14) | 1.10 (0.82-1.48) | **1.13 (1.01-1.27)** | 1.12 (0.82-1.53) |  |
| **Sex** |  |  |  |  |  |
| Female | — | — | — | — |  |
| Male | **0.94 (0.92-0.96)** | **0.91 (0.86-0.98)** | **0.56 (0.54-0.57)** | **0.64 (0.59-0.68)** |  |
| **Age** |  |  |  |  |  |
| 25-34 | — | — | — | — |  |
| 18-24 | **0.83 (0.80-0.86)** | **0.86 (0.78-0.94)** | **0.48 (0.46-0.50)** | **0.55 (0.50-0.61)** |  |
| 35-44 | **1.15 (1.12-1.18)** | 1.05 (0.97-1.14) | 1.02 (0.99-1.06) | **1.16 (1.06-1.27)** |  |
| 45-54 | **1.16 (1.13-1.20)** | **1.12 (1.03-1.22)** | **1.17 (1.13-1.21)** | **1.36 (1.25-1.49)** |  |
| 55-64 | **1.20 (1.16-1.25)** | **1.22 (1.11-1.34)** | **1.50 (1.44-1.56)** | **1.55 (1.39-1.72)** |  |
| 65-67 | 1.06 (0.97-1.15) | 0.87 (0.64-1.19) | 0.92 (0.84-1.02) | **0.47 (0.34-0.64)** |  |
| **Educational level** |  |  |  |  |  |
| College/university | — | — | — | — |  |
| High school | **1.05 (1.02-1.09)** | 1.04 (0.95-1.13) | **1.46 (1.41-1.51)** | **1.33 (1.22-1.44)** |  |
| Elementary | **1.13 (1.09-1.18)** | **1.15 (1.04-1.27)** | **1.69 (1.62-1.76)** | **1.50 (1.35-1.67)** |  |
| **Family situation** |  |  |  |  |  |
| Single without children (<18 years) at home | — | — | — | — |  |
| Single with children at home | **1.06 (1.01-1.10)** | 1.03 (0.92-1.17) | **1.48 (1.40-1.56)** | **1.30 (1.13-1.51)** |  |
| Married/cohabiting without children at home | **0.95 (0.92-0.98)** | 0.92 (0.84-1.00) | **1.04 (1.00-1.08)** | 1.06 (0.96-1.17) |  |
| Married/cohabiting with children at home | **0.90 (0.88-0.92)** | **0.91 (0.85-0.97)** | **1.24 (1.21-1.28)** | 1.01 (0.93-1.09) |  |
| **Region of birth** |  |  |  |  |  |
| Sweden | — | — | — | — |  |
| Nordic countries (except Sweden) | 0.97 (0.89-1.05) | 1.11 (0.87-1.42) | **1.11 (1.01-1.22)** | 1.11 (0.86-1.42) |  |
| EU27 (except Denmark, Finland, and Sweden) | **0.91 (0.86-0.97)** | 0.92 (0.77-1.08) | **1.08 (1.01-1.16)** | 1.10 (0.91-1.32) |  |
| Rest of the world | **0.96 (0.93-0.99)** | 0.92 (0.85-1.01) | **1.09 (1.05-1.12)** | **1.33 (1.22-1.46)** |  |
| **Type of place of residence^c^** |  |  |  |  |  |
| Large city | — | — | — | — |  |
| Medium-size town | 0.99 (0.97-1.02) | 1.01 (0.95-1.08) | 1.00 (0.97-1.02) | 0.97 (0.91-1.03) |  |
| Small town or rural area | 1.01 (0.98-1.04) | 1.03 (0.95-1.11) | 1.02 (0.99-1.05) | 0.93 (0.85-1.01) |  |
| ^a^ The Count component estimates the average number of SA net days, amongst individuals who had some SA (i.e., >0 SA net days), using a truncated negative binomial model. ^b^ The Hurdle component estimates the odds of having any SA, using a logistic regression model. ^c^ Defined according to Eurostat’s Degree of Urbanisation (DEGURBA) (22) | | | | |  |
