# Supplementary material for: Associations between prior and subsequent sickness absence before and during the COVID-19 pandemic: a Swedish prospective cohort study of 306 933 blue-collar workers in the retail and wholesale industry
Source: BMJ Open. 2025 Oct 27;15(10):e096764. doi: 10.1136/bmjopen-2024-096764 (PMC12581036; doi:10.1136/bmjopen-2024-096764)
Supplement: Supplementary file 2 [file bmjopen-2024-096764.reviewer_comments.pdf]

PEER REVIEW HISTORY

BMJ Open publishes all reviews undertaken for accepted manuscripts. Reviewers are asked to complete a checklist review form and are provided with free text boxes to elaborate on their assessment. These free text comments are reproduced below.

ARTICLE DETAILS

Title (Provisional)

Associations between prior and subsequent sickness absence before and during the COVID-19 pandemic: a Swedish prospective cohort study of 306 933 blue-collar workers in the retail and wholesale industry

Authors

Cybulski, Lukasz; Pettersson, Emma; Alexanderson, Kristina; Farrants, Kristin

---

VERSION 1 - REVIEW

Reviewer 1

Name Nwaru, Chioma

Affiliation University of Gothenburg, School of Public Health and Community Medicine, Institute of Medicine, Sahlgrenska Academy

Date 18-Feb-2025

COI None

The manuscript investigated associations between prior and subsequent sickness absence before and during the COVID-19 pandemic among blue-collar workers in retail and wholesale industries. The paper is generally well-written, the database is valued, and the research question is very relevant. I have only a few minor comments.

The manuscript investigated associations between prior and subsequent sickness absence before and during the COVID-19 pandemic among blue-collar workers in retail and wholesale industries. The paper is generally well-written, the database is valued, and the research question is very relevant. I have only a few minor comments.

Title

1. line 5: did you mean semi-colon or colon?
2. The spelling of Covid-19 is different from the WHO recommendation.

Abstract

1. Lines 17-18 “we examined how these patterns varied according to occupation, sociodemographic indicators, and the length and frequency of SA in the preceding year”. Is this a secondary aim because there is no mention of this in the results section of both the abstract and the main text?

2. Method section could include data source (register or survey).

Methods

1. Line 58: COVID-19- missing a hyphen?
2. Line 16: aged 18-67 years (also check other sections of the manuscript).
3. Lines 16-17: "the study population of individuals aged ...... living in Sweden on 31 December in both 2018 and 2019". Are these the baseline dates for both periods (i.e., 2018-2019 and 2020-2021?
4. Line 26: "Individuals were excluded from the analysis if they were not resident in Sweden when SA exposures were measured…? Would this be in 2017 and 2019?

Results

1. What is the mean age of the participants?
2. Table 1: how is baseline defined?
3. I would add a definition of place of residence as a footnote.
4. Table 3: I would add 'adjusted associations…' in the title to enhance clarity.
5. Rather than including the estimates for the covariates in Table 3, I would merge Tables 3 and SI (i.e., crude and adjusted results) and maybe provide the results for the covariates as a supplementary file.
6. It is unclear why crude and adjusted estimates for the covariates were provided.

Discussion

1. Line 42: "of across…"?

**Reviewer**	**2**
**Name**	**Dack, Kyle**
**Affiliation**	**University of Bristol, MRC Integrative Epidemiology Unit**
**Date**	**05-Aug-2025**
**COI**	**None**

Statistical review summary

The study is well designed, choosing suitable statistical methods and reporting results clearly. I have no major concerns regarding the statistical analysis or its interpretation. Below are minor points of clarification which would enhance the paper.

Abstract

1. Clear – no suggestions.

Introduction

2. The introduction is clear and well written. However, one question I had which it did not answer, is why the study focused on SA of 15 or more days. The introduction does not distinguish between short-term and long-term SA, but the study design focused on long-term (>=15 days) SA only.

Methods

3. Study design: in the first instance of reporting the dates, it would help readers if the full date was stated, because 2018-2019 could mean quite different lengths depending on the specific months.

4. Related to the previous comment, the section titled “Public Sickness Absence Insurance in Sweden” is where it is first stated that the study is limited to SA spells of 15 days or longer. However it is unclear to me whether this was a decision by the authors based on the research question, or a limitation of the available data. I recommend clarifying this either in the introduction or methods. Especially if one of the outcomes of interest is length of SA, as this decision puts a floor of 15 days on any identified SA.

5. To what extent is there a risk of employers terminating those on extremely long-term SA, which would impact the detected lengths? It may be helpful to discuss for readers unfamiliar with the employment system in Sweden, perhaps in the discussion.

6. Outcomes: “The exposure of SA was categorised into four groups: 0 days, 1-30” – should “1-30” not be “15-30”? The prior section stated “In this study, only information on SA spells lasting longer than 14 days were used in the computation of the outcome and exposure measures” which should mean that values of 1-14 are not possible?

7. Typo: “Information on the following sociodemographic variables were included as covariates in the analysis: sex; age (18-4” should be 18-24.

8. Statistical analysis: I confirm I have no concerns regarding the stats methods used or their reporting – a suitable model was selected and described clearly.

Results

9. I suggest considering adding a histogram of the outcome SA days for each time period, to help readers understand visually how SA was distributed. This would also help to support model selection – a justification of using the negative binomial hurdle regression was overdispersion but currently readers cannot see this overdispersion or statistics which demonstrate it.

10. Table 1. The age groups are slightly out of order – the first two rows should be swapped.

11. Table 2, 3, 4. Unless I have misinterpreted how SA was handled, I suggest changing the group labelled as SA: 1-30 days as 15-30, because no individuals had values of 1-14.

Discussion

12. The study reports the 14 day SA restriction as a limitation, and it sounds as if it may have been a data availability issue rather than design preference. If so I suggest clarifying this in the methods. I suggest this could be elaborated on in terms of what impact it has on the statistics. It is a major caveat for certain findings like the median number of SA days. But I am unsure of the implications in terms of the primary model results, and if the authors could describe this in more detail it would help readers understand the implication of this caveat.

VERSION 1 - AUTHOR RESPONSE

#	Reviewer comment	Author response
	Reviewer 1	
	**Title**	
1	line 5: did you mean semi-colon or colon?	We've now changed this to a colon. Thanks.
2	The spelling of Covid-19 is different from the WHO recommendation	We now consistently use the term COVID-19 throughout the manuscript, which is the term preferred by the World Health Organization.
	**Abstract**	
3	Lines 17-18 "we examined how these patterns varied according to occupation, sociodemographic indicators, and the length and frequency of SA in the preceding year". Is this a secondary aim because there is no mention of this in the results section of both the abstract and the main text?	Thanks for alerting us on that this was not clear. The examination of occupation and sociodemographic factors as predictors for SA was not the primary aim of the study. We have already examined how these factors were associated with SA during the pandemic in a related manuscript that has been published elsewhere.<br><br>Because we did not discuss the estimates for occupation and sociodemographic factors in the results or discussion section of the manuscript, we have opted to remove them. Interested readers can now find these results in the supplementary materials (Table S2 and S3). We think this revision also is in line with suggestions made in comments 12 and 13. We now briefly comment on the associations related to occupation and sociodemographic factors in the results section on page 7 and refer the reader to the supplementary materials:<br><br>"Occupation and sociodemographic factors were generally less strongly associated with the duration and likelihood of SA (Table S2 & S3)."<br><br>We have also made corresponding changes to the abstract. The portion of the abstract the reviewer highlighted now reads:<br><br>"We examined how these patterns varied according to the length and frequency of SA in the preceding year"

4	Method section could include data source (register or survey).	We have a dedicated section to this on page 4 under the heading "Data and study population". This section opens with a sentence that indicates that the data were derived from a several interlinked registers:<br><br>"Individual-level pseudonymised data from three nationwide Swedish administrative registers, linked by personal identification numbers (18) were used in order to delineate the study population and construct study outcomes and covariates."
	**Methods**	
5	Line 58: COVID-19- missing a hyphen	Thank you.
6	Line 16: aged 18-67 years (also check other sections of the manuscript).	We've now added "years" as the reviewer suggests.
7	Lines 16-17: "the study population of individuals aged …… living in Sweden on 31 December in both 2018 and 2019". Are these the baseline dates for both periods (i.e., 2018-2019 and 2020-2021?	Those dates are the follow-up periods before (2018-2019) and during (2020-2021) the pandemic. The corresponding baseline dates for these periods are 2017 and 2019, respectively.<br><br>We now describe this in detail in the "Outcomes, Exposures, and Covariates" section on page 5:<br><br>"The outcome measure was the number of SA days in the two time periods: before the COVID-19 pandemic (01 January 2018 - 31 December 2019) and during the pandemic (01 January 2020 - 31 December 2021). The primary exposure was the number of SA days, measured the calendar year before each of these two periods, i.e., in 2017 and in 2019, respectively."
8	Line 26: "Individuals were excluded from the analysis if they were not resident in Sweden when SA exposures were measured…? Would this be in 2017 and 2019?	That is correct. We have modified the sentence to enhance clarity:<br><br>"Individuals were excluded from the analysis if they were not resident in Sweden when SA exposures were measured in 2017 and 2019 (n=119)..."
	**Results**	
9	What is the mean age of the participants?	• Mean (SD): 35.32 (13.13)<br>• Median (IQR): 31 (24-45)<br><br>We have now added information about the mean age to the results section on page 6:<br><br>"Of the included 306 933 blue-collar workers in the retail and wholesale industry, 57.2% were aged

		18-34 and 47.9% were women (Table 1). The mean age was 35.2 (standard deviation: 13.1)."
10	Table 1: how is baseline defined?	The data presented in Table 1 is from 2019. We have now added the year in the title of the table to clarify this:<br><br>"**Table 1**. Cohort characteristics in 2019 among blue-collar workers in retail and wholesale"
11	I would add a definition of place of residence as a footnote.	We have added a footnote to tables 1, 3, S1, S2, and S3:<br><br>"Defined according to Eurostat's Degree of Urbanisation (DEGURBA) (22)"
12	Table 3: I would add 'adjusted associations...' in the title to enhance clarity.	Thank you, we have implemented this suggestion. Since we have also added the crude estimates to this table on the advice of the other reviewer, the title now reads "crude and adjusted associations…"
13	Rather than including the estimates for the covariates in Table 3, I would merge Tables 3 and SI (i.e., crude and adjusted results) and maybe provide the results for the covariates as a supplementary file	We have redrawn our tables and moved all estimates relating to the covariates (e.g., sociodemographic factors and occupation) to the supplementary materials. We now end the results section with a brief summary and refer the reader to tables S2 and S3 for the covariates. We have added the following sentence to the end of the results section on page 7:<br><br>"Occupation and sociodemographic factors were generally less strongly associated with the duration and likelihood of SA (Table S2 & S3)"<br><br>Table 3 now contains crude and adjusted estimates regarding prior SA length and future length and likelihood of SA. Table S2 contains the crude associations between occupation and sociodemographic factors and the length and likelihood of SA, whereas S3 contains the adjusted estimates, which previously were in Figure 3.
14	It is unclear why crude and adjusted estimates for the covariates were provided.	Please see our response to comment #12.
	**Discussion**	
15	Line 42: "of across..."?	Thank you. We have removed "of".
	**Reviewer 2**	

	**Introduction**	
16	The introduction is clear and well written. However, one question I had which it did not answer, is why the study focused on SA of 15 or more days. The introduction does not distinguish between short-term and long-term SA, but the study design focused on long-term (>=15 days) SA only.	For employed individuals who are sickness absent, the employer covers sick pay during the first 14 days of a sickness absence spell. After 14 days, sickness absence benefits are issued by the National Social Insurance Agency. Because we utilised data from the Social Insurance Agency, we do not have information on spells which fall outside of their purview (i.e., SA spells shorter than 15 days). There is no general information in Sweden about the shorter SA spells. We now discuss this as a limitation in the strength and limitations section on page 9:<br><br>"Our study also had some limitations. For instance, we could only study SA spells that exceed 14 days, as spells below this duration are outside the purview of the Social Insurance Agency."
	**Methods**	
17	Study design: in the first instance of reporting the dates, it would help readers if the full date was stated, because 2018-2019 could mean quite different lengths depending on the specific months.	Under the study design heading on page 3-4 we now write:<br><br>"Measures of SA before (01 January 2018 - 31 December 2019) and during the COVID-19 pandemic (01 January 2020 - 31 December 2021) were compared"<br><br>And later in the Outcomes, Exposures, and Covariates section on page 5:<br>"The outcome measure was the number of SA days in the two time periods: before the COVID-19 pandemic (01 January 2018 - 31 December 2019) and during the pandemic (01 January 2020 - 31 December 2021)."
18	Related to the previous comment, the section titled "Public Sickness Absence Insurance in Sweden" is where it is first stated that the study is limited to SA spells of 15 days or longer. However it is unclear to me whether this was a decision by the authors based on the research question, or a limitation of the available data. I recommend clarifying this either in the introduction or methods. Especially if one of the outcomes of interest is length of SA, as	We also provided this information in the final sentence of method section of the abstract: "Only SA spells >14 days were included."<br><br>We have also added a sentence to the "Public Sickness Absence Insurance in Sweden" section on page 4 to clarify why we restricted ourselves to SA spells longer than 14 days.<br><br>"In this study, only information on SA spells lasting longer than 14 days were used in the computation of the outcome and exposure

	this decision puts a floor of 15 days on any identified SA	measures. This is because SA spells below this duration are not recorded by the Social Insurance Agency.”
19	To what extent is there a risk of employers terminating those on extremely long-term SA, which would impact the detected lengths? It may be helpful to discuss for readers unfamiliar with the employment system in Sweden, perhaps in the discussion.	Individuals who are unemployed may also be sickness absent. Being on unemployment benefits qualifies individuals for sickness absence benefits if they are unable to seek employment due to illness or injury. Thus, even if a person were to lose their employment during a SA spell, it would not necessarily mean that they would stop receiving SA benefits.<br><br>With regards to your question about to what extent there is a risk the employers terminate the contracts of those on extremely long-term SA: it is very low. By Swedish law, employers cannot terminate an employee’s contract because of sickness absence. However, individuals who are on SA may lose their job for other reasons. Employers are not obliged to extend fixed-term contracts of employees should they miss extended periods of work due to sickness absence, and if there are general lay-offs at a company, sickness absent employees are treated in the same way as other employees in deciding who has priority to remain. If individuals become unemployed during their sickness absence, their work capacity is assessed in relation to any work on the regular labour market for the continuation of SA benefits from the day they become unemployed, and the Social Insurance Agency may withdraw SA benefits if they deem that the person has the work capacity for another job.<br>After day 180 of a SA spell, the work capacity and thus the right to continued SA benefits of all sickness absent individuals is assessed in relation to any work on the regular labour market in the same way as for unemployed people.<br><br>In sum, there is very little risk that a change to a person’s employment status would have an influence on how complete data is vis-à-vis sickness absence: the only situation in which a change in job status would influence the length of SA is if someone lost their job (unrelatedly to their SA) during the first 180 days of their SA spell, and was then deemed to be able to take on any regular job, when their work capacity would otherwise be

		assessed against their own previous work or other work at the same employer had they retained their job.<br><br>We have added a sentence (in bold below) to clarify that unemployment will not lead to an underreporting of SA under the heading "Public Sickness Absence Insurance in Sweden" in the method section on page 4:<br><br>"In Sweden, individuals with income from work, parental-leave, or unemployment benefits, can claim SA benefits if their work capacity is reduced due to morbidity. **Because individuals who are unemployed also qualify for SA benefits, there is no risk of under-recording SA if individuals become unemployed after or during a SA spell.** After a first waiting day, employers pay SA benefits during the subsequent 13 days of a SA spell. Thereafter, payments are covered by the Social Insurance Agency (the Social Insurance Agency pays from day 2 for unemployed people)."
20	Outcomes: "The exposure of SA was categorised into four groups: 0 days, 1-30" – should "1-30" not be "15-30"? The prior section stated "In this study, only information on SA spells lasting longer than 14 days were used in the computation of the outcome and exposure measures" which should mean that values of 1-14 are not possible?	We have added an explanation in the method section on page 5:<br><br>"Whilst we could not access data on SA spells below 15 days, we nevertheless included a "1-30 days" category for our SA exposure measure. This is because some SA spells will have started before the exposure periods (e.g., in 2016) and continued into the next year and then ended shortly thereafter (e.g., beginning of 2017). In these cases, individuals may have had less than 14 days of SA in the exposure period (e.g., 2017), but more than 15 days across both 2016 and 2017. Similarly, SA spells that began shortly before the end of an exposure period (e.g., 28 December 2017) but which continued for more than 15 days into the next year will also have been in this category."
21	Typo: "Information on the following sociodemographic variables were included as covariates in the analysis: sex; age (18-4" should be 18-24.	Thank you.
	**Results**	
22	I suggest considering adding a histogram of the outcome SA days for each time period, to help readers	We have now added a histogram ("**Figure S1.** Distribution of observations according to the number of net SA days by observation period.") to

	understand visually how SA was distributed. This would also help to support model selection – a justification of using the negative binomial hurdle regression was overdispersion but currently readers cannot see this overdispersion or statistics which demonstrate it.	the supplementary materials that we reference in our description of the statistical methods on page 6:<br><br>"This is a two-component model that is appropriate when analysing over-dispersed count data with an excess of zero counts (24) (please see Figure S1 in the supplementary materials)."
23	Table 1. The age groups are slightly out of order – the first two rows should be swapped.	We have deliberately placed the second youngest group first in the order since they are the largest group and therefore the reference group.
24	Table 2, 3, 4. Unless I have misinterpreted how SA was handled, I suggest changing the group labelled as SA: 1-30 days as 15-30, because no individuals had values of 1-14.	Please see our response to comment #20.
	**Discussion**	
25	The study reports the 14 day SA restriction as a limitation, and it sounds as if it may have been a data availability issue rather than design preference. If so I suggest clarifying this in the methods. I suggest this could be elaborated on in terms of what impact it has on the statistics. It is a major caveat for certain findings like the median number of SA days. But I am unsure of the implications in terms of the primary model results, and if the authors could describe this in more detail it would help readers understand the implication of this caveat.	As the reviewer says, this is a limitation imposed on us by the data. We have added a couple of sentences in the discussion on page 9 to reflect on some of the implications of this:<br><br>"For instance, we could only study SA spells that exceed 14 days. Whilst these longer spells account for the majority of the total number of SA days in the population, many SA spells are shorter (33). Thus, SA spells that lasted for less than 15 days will not have been included in this study. Whilst our study demonstrated only a slight increase in SA during the pandemic, it is conceivable that the pattern might have been different for shorter spells."
